# Real-time Characterization of the Morphology of High-Performance Engineering Thermoplastics During Additive Manufacturing via Synchrotron X-ray Diffraction

**DOI:** 10.1063/4.0001031

**Published:** 2025-10-27

**Authors:** Kirt Page, Jacob Crossno, Mia Carrola, Devin Ryan, Hilmar Koerner, Arthur Woll, Louisa Smieska

**Affiliations:** 1 BlueHalo, Dayton, Ohio, USA; 2 Air Force Research Laboratory, Dayton, Ohio, USA; 3 Cornell High Energy Synchrotron Source, Ithaca, New York, USA

## Abstract

Additively manufactured, high-performance engineering thermoplastics are increasingly used to realize complex, 3-dimensional (3D) products. However, abrupt changes in material properties at the interface between printed roads can lead to poor performance. More specifically, heterogeneities in morphology that develop during the printing process can lead to poor interfacial adhesion between printed roads and, therefore, poor mechanical performance. To gain insight on the origins of heterogeneities in 3D-printed parts, we report a combination of synchrotron-based *in-situ* X-ray microdiffraction and infrared pyrometry to map the formation and evolution of crystallinity in polyether ether ketone (PEEK) in a series of prints. The spatio- temporal maps of crystallinity created from the X-ray data coupled with the variation in temperature through the height of the printed layers, as obtained from the infrared pyrometry data, has enabled the obtainment of a detailed description of the crystallization behavior. The crystallinity maps and analysis of the crystallization kinetics reveal the development of heterogeneities within and between layers of printed PEEK and provides insights into the non-equilibrium process of 3D printing.

